# Neuroprotection Against NMDA-Induced Retinal Damage by Philanthotoxin-343 Involves Reduced Nitrosative Stress

**DOI:** 10.3389/fphar.2021.798794

**Published:** 2021-12-14

**Authors:** Mohamad Haiqal Nizar Mohamad, Izuddin Fahmy Abu, Muhammad Fattah Fazel, Renu Agarwal, Igor Iezhitsa, Norsham Juliana, Ian R. Mellor, Henrik Franzyk

**Affiliations:** ^1^ Institute of Medical Science Technology, Universiti Kuala Lumpur, Kuala Lumpur, Malaysia; ^2^ School of Medicine, International Medical University, Kuala Lumpur, Malaysia; ^3^ Department of Pharmacology and Bioinformatics, Volgograd State Medical University, Volgograd, Russian Federation; ^4^ Faculty of Medicine and Health Sciences, Universiti Sains Islam Malaysia, Negeri Sembilan, Malaysia; ^5^ School of Life Sciences, Faculty of Medicine and Health Sciences, University of Nottingham, Nottingham, United Kingdom; ^6^ Department of Drug Design and Pharmacology, Faculty of Health and Medical Sciences, University of Copenhagen, Copenhagen, Denmark

**Keywords:** excitotoxicity, neurodegeneration, N-methyl-D-aspartate receptor, N-methyl-D-aspartate receptor overstimulation, philanthotoxin-343, retinal ganglion cell, visual behavioural analysis, glaucoma

## Abstract

N-methyl-D-aspartate receptor (NMDAR) overstimulation is known to mediate neurodegeneration, and hence represents a relevant therapeutic target for neurodegenerative disorders including glaucoma. This study examined the neuroprotective effects of philanthotoxin (PhTX)-343 against NMDA-induced retinal injury in rats. Male Sprague Dawley rats were divided into three groups; group 1 received phosphate buffer saline as the negative control, group 2 was injected with NMDA (160 nM) to induce retinal excitotoxic injury, and group 3 was pre-treated with PhTX-343 (160 nM) 24 h before NMDA exposure. All treatments were given intravitreally and bilaterally. Seven days post-treatment, rats were subjected to visual behaviour assessments using open field and colour recognition tests. Rats were then euthanized, and the retinas were harvested and subjected to haematoxylin and eosin (H&E) staining for morphometric analysis and 3-nitrotyrosine (3-NT) ELISA protocol as the nitrosative stress biomarker. PhTX-343 treatment prior to NMDA exposure improved the ability of rats to recognize visual cues and preserved visual functions (i.e., recognition of objects with different colours). Morphological examination of retinal tissues showed that the fractional ganglion cell layer thickness within the inner retina (IR) in the PhTX-343 treated group was greater by 1.28-fold as compared to NMDA-treated rats (*p* < 0.05) and was comparable to control rats (*p* > 0.05). Additionally, the number of retinal cell nuclei/100 μm^2^ in IR for the PhTX-343-treated group was greater by 1.82-fold compared to NMDA-treated rats (*p* < 0.05) and was comparable to control group (*p* > 0.05). PhTX-343 also reduced the retinal 3-NT levels by 1.74-fold compared to NMDA-treated rats (*p* < 0.05). In conclusion, PhTX-343 pretreatment protects against NMDA-induced retinal morphological changes and visual impairment by suppressing nitrosative stress as reflected by the reduced retinal 3-NT level.

## Introduction

Glaucoma is one of the world’s leading causes of irreversible blindness (Banerjee et al., 2019) affecting more than 70 million individuals globally (Yang and Weinreb, 2019). It is characterized by degenerative changes in the optic nerve and retinal ganglion cell (RGC) death leading to irreversible visual field loss (Quigley and Broman, 2006; Tham et al., 2014; Jafri et al., 2018; Banerjee et al., 2019). It has a multifactorial aetiology and various risk factors such as genetics, race, age and cardiovascular diseases, which contribute to its onset and progression (Coleman and Miglior, 2008). Elevated intraocular pressure (IOP) is recognized as a major risk factor in the development of glaucoma, hence, lowering the IOP is currently the focus of the disease management. However, glaucomatous changes often progress despite IOP lowering and appear in patients even with normal IOP (Kumar and Agarwal, 2007). Current antiglaucoma medications are also associated with several adverse reactions such as conjunctival hyperemia, ocular surface disease, and ocular allergy, as well as systemic effects, including bradycardia, irregular pulse and aggravation of bronchial asthma (Cvenkel and Kolko, 2020). All the adverse reactions in turn, often result in poor patient compliance (Inoue, 2014). These issues highlight the insufficiency of current management strategies, hence, new therapies that can protect against RGC apoptosis, with or without IOP reduction, will be valuable for arresting disease progression.

Apoptotic loss of RGC is a pathophysiological hallmark of glaucoma (El-Danaf and Huberman, 2015; Yohannan and Boland, 2017), and hence it is logical to investigate new drugs that can target the pathophysiological mechanisms leading to RGC loss in the presence or absence of elevated IOP. One such mechanism that triggers RGC loss regardless of IOP level is glutamate-mediated excitotoxicity (Dong et al., 2009; Christensen et al., 2019; Vidal-Villegas et al., 2019). Excessive glutamatergic transmission is known to damage RGC selectively in mammalian eyes (Lambuk et al., 2017; Milla-Navarro et al., 2021). Among various glutamate receptors the N-methyl-D-aspartate receptor (NMDAR) is particularly implicated in excitotoxic retinal damage as its activation triggers calcium influx, excessive nitric oxide (NO) production, and activation of apoptotic pathways leading to subsequent neurodegenerative changes in the retina (Chen et al., 1992; Doozandeh and Yazdani, 2016; Rosini et al., 2019).

In recent years, natural and synthetic bioactive compounds have been intensively explored and studied for their therapeutic potential against RGC loss in glaucomatous animal models (Lambuk et al., 2017; Chou et al., 2018; Jafri et al., 2018; Lambuk et al., 2019a; Jafri et al., 2019; Chou et al., 2020; Fazel et al., 2020; Jafri et al., 2020; Romano et al., 2020; Conti et al., 2021). Philanthotoxin (PhTX) is a potent NMDAR modulator (Piek, 1982) representing a relevant therapeutic option to counteract excitotoxicity and prolong RGC survival in glaucoma. PhTX-433 is the pure natural toxin, isolated from solitary digger wasp, *Philanthus triangulum,* comprised of a butyryl/tyrosyl/polyamine structure. It has been shown to block excitatory ion channels such as nicotinic acetylcholine receptors (nAChRs) and ionotropic glutamate receptors (iGluRs) (Kachel et al., 2016). A variety of synthetic PhTX analogues have been developed where structural modifications have been shown to improve affinity and selectivity of the compounds for NMDAR (Mellor et al., 2003; Strømgaard et al., 2005; Frølund et al., 2010; Kachel et al., 2019; Banke and Barria, 2020). PhTX-343 is one of the synthetic analogues that is structurally similar to PhTX-433 (Kachel et al., 2019), and it retains the active pharmacological properties of the parent compound (Eldefrawi et al., 1988; Fazel et al., 2020). In the PhTX-343 molecule, the thermospermine tail (polyamine component) has been exchanged for the symmetrical spermine. Like the original compound, this synthetic analogue was shown to exhibit non-competitive antagonistic properties for all three classes of iGluRs including NMDAR (Huang et al., 1997; Bähring and Mayer, 1998; Kachel et al., 2019). Hence, the present study was conducted to assess the neuroprotective effects and mechanism of action of PhTX-343 against NMDA-induced excitotoxic retinal damage to preserve visual functions.

## Materials and Methods

### Animal Preparation

Male Sprague Dawley rats weighing 200–250 g and aged between 50–55 days were used in this study. The rats were housed under standard laboratory conditions with a reverse light-dark cycle of 12 h:12 h with access to food and water ad libitum.

All animals found normal on systemic and ophthalmic examinations were included in this study and were acclimatized for 1 week prior to daily handling. During acclimatization and treatment period, animals were also habituated to handling gradually prior to behavioural assessments. All use and handling of animals complied with the principles set by the Association for Research in Vision and Ophthalmology (ARVO) and prior ethical approval was obtained from the Animal Ethics Committee, Institute of Medical Science Technology, Universiti Kuala Lumpur (approval number: AEC/MESTECH-UNIKL/2018/001) and Institutional Animal Ethics Committee (UiTM CARE), Universiti Teknologi MARA (approval number: UITM CARE:239/2/2018 (April 6, 2018)).

### Study Design

The rats were divided into three groups, each consisting of 12 rats (24 eyes). Prior to administering the treatments, animals were anaesthetized through intraperitoneal administration of ketamine (80 mg/kg) and xylazine (12 mg/kg) mixture (Ilium Troy Laboratories, Glendenning, New South Wales, Australia). All treatments were administrated via intravitreal injection in both eyes (Lambuk et al., 2019b; Fazel et al., 2020). Rats in group 1 were intravitreally injected with phosphate buffer saline (PBS) (Sigma-Aldrich, St. Louis, MO, United States ) and served as the negative control group. Group 2 rats were injected with NMDA (Sigma-Aldrich) at a concentration of 160 nM to induce retinal excitotoxic injury (Lambuk et al., 2019a; Fazel et al., 2020). Rats in group 3 were treated with PhTX-343, 24 h prior to NMDA administration as the experimental group. PhTX-343 was synthesized at the University of Copenhagen, Denmark and was also administered at an equimolar concentration of 160 nM in PBS (pH7.4).

**FIGURE 1:**
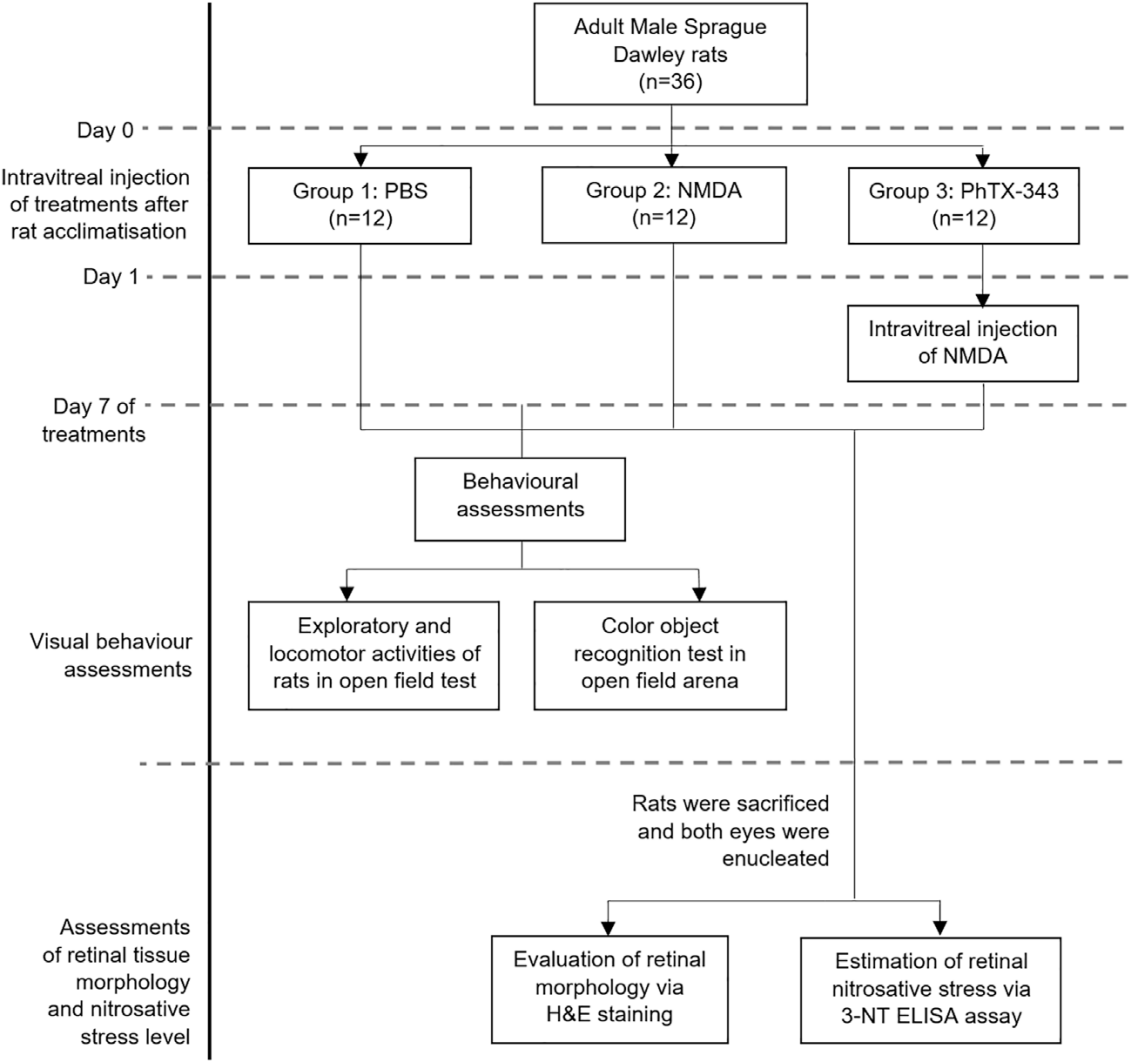
Overall study design of the project. PBS, phosphate buffer saline; NMDA, N-methyl-D-aspartate; PhTX-343, philanthotoxin-343; 3-NT, 3-nitrotyrosine.

For all treatments, 2 μL volume was administered in the rat eyes assisted by a dissecting microscope. The intravitreal injection was performed by using a 26-gauge 10 μL syringe (Hamilton, Reno, Nevada, United States ) through sclera at 1 mm from the limbus in superotemporal quadrant. The procedure was conducted slowly over 2 min to prevent pressure-induced retinal damage. Neomycin and Polymyxin B Sulfates ophthalmic suspension (Bausch + Lomb, Quebec, Canada) were applied post-injection (Fazel et al., 2020). After 7 days of treatment, the rats were subjected to visual behaviour tests which were conducted between 8.00 and 10.00 am, during which the animals demonstrate the highest behavioural activities, and then were sacrificed. The eyes were enucleated for retinal morphology assessments using hematoxylin and eosin (H&E) staining and retinal nitrosative stress level using 3-nitrotyrosine (3-NT) ELISA kit (Elabscience, Wuhan, China). The overall study design is illustrated in Figure 1.

### Assessment of Rat Exploratory Behaviour Using Open Field Test

In this experiment, the open field test was utilized to evaluate the exploratory and anxiety-related behaviour of animals (Fazel et al., 2020; Mohd Lazaldin et al., 2020). The open field mechanical assembly consisted of PVC square arena of 100 cm (L) × 70 cm (H) × 100 cm (W) and was set up in a soundproof room. The entire open field arena was divided into “peripheral” (10,000 cm^2^ every 100 cm (L) × 100 cm (W)) and “central” (2,500 cm^2^ every 50 cm (L) × 50 cm (W)) zones, and was lit up with a 100-W tungsten light which has a UV-absorbing glass filter around the bulb to filter UV-radiation. A camera was positioned above the central part of the open field arena. Each rat was placed at the centre and was allowed to explore the open field arena for 10 min (trial 1) during the habituation stage. This was followed by introduction of colour objects in the open field arena (trials 2–4) for further exploratory behaviour assessments and colour recognition test (further described in 2.4). All movements and behavioural reactions of the rats were recorded and analyzed using ANY-maze software (Stoelting Co., Wood Dale, Illinois, United States ). The parameters measured included the total distance travelled, total immobile time and total immobile episodes. The floor of the open field was wet-cleaned with 75% ethanol (HmbG Chemicals, Hamburg, Germany) before each trial to avert transmission of olfactory cues.

### Assessment of Colour Object Recognition using Open Field Arena

**FIGURE 2:**
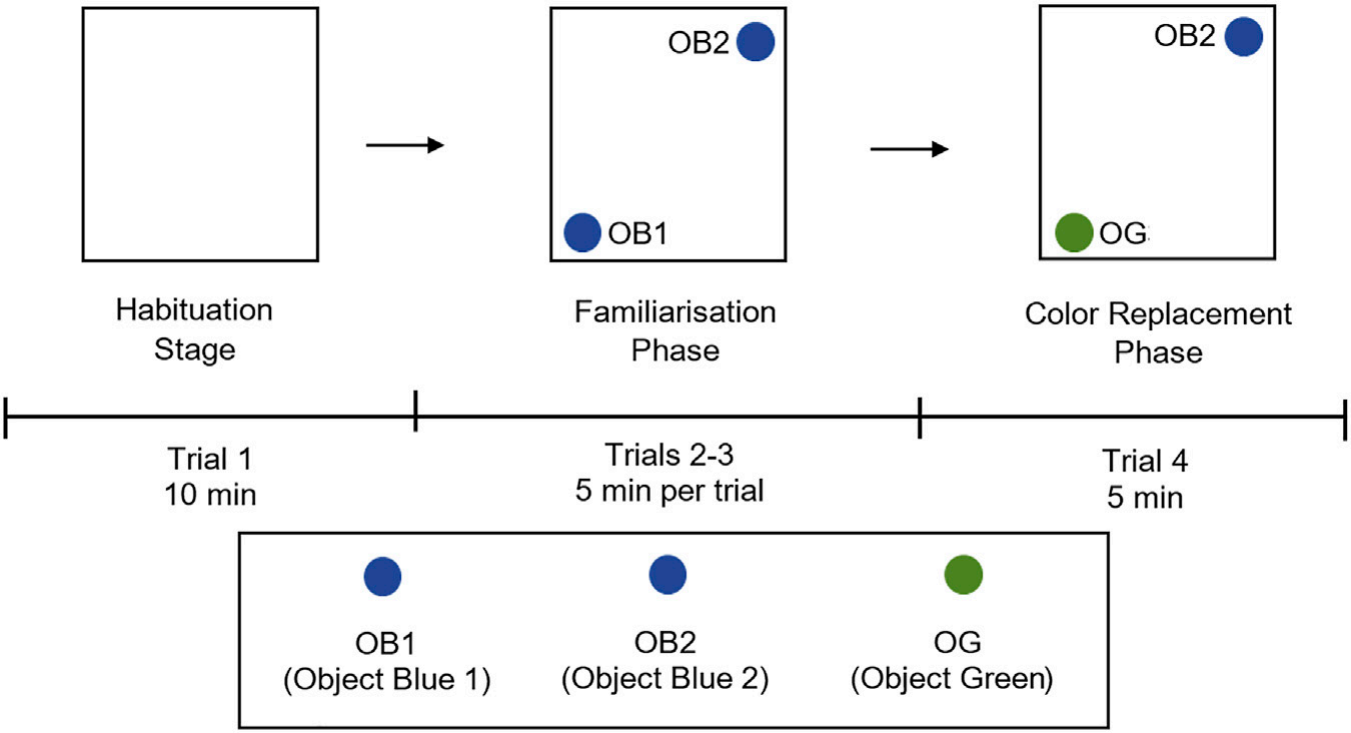
Experimental design and placement of objects for colour object recognition test in open field arena. The experimental design has three phases; habituation stage (trial 1) with empty open arena, familiarization phase (trials 2 and 3) with two blue colour objects (OB1;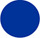 and OB2;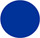) in the arena, and colour replacement phase (trial 4) where one blue object (OB1;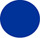) is replaced with a green object (OG;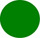). Rats were placed at the centre of the arena alone and allowed to explore freely, the rat’s movements were recorded using Any-maze software. OB1, object blue 1; OB2, object blue 2, OG, object green.

The colour recognition test was adopted with slight modification to previously described protocols (Zhang et al., 2012; Jafri et al., 2020). These tests (trials 2–4) were conducted to assess the effects of PhTX-343 on NMDA-induced changes on colour perception by rats. In this experiment, three coloured cylindrical glass bottles with rounded ends were used, and these were of similar height; 15 cm in average, to avoid any bias. Objects 1 and 2 (OB1;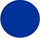 and OB2;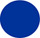) were blue in colour while object 3 was green in colour (OG3;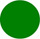). All coloured objects are equiluminance. Firstly, the two blue colour objects (OB1;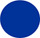 and OB2;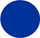) were positioned diagonally in the arena, followed by placing the rats at the centre. The rats were then allowed to explore the arena freely. This familiarization stage consisted of two trials (2 and 3) lasting for 5 minutes each to allow rats to develop spatial memory. Subsequently, one of the blue object (OB1;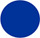) was replaced with a green colour object (OG3;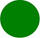). The rats were then allowed to explore the arena containing one blue object (OB1;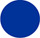) and the newly introduced green object (OG3;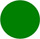) for 5 minutes. All movements and behavioural responses by the rats were recorded and analyzed using ANY-maze software (Stoelting Co.) To avoid olfactory cues, the open field arena was wet-mopped with 75% ethanol solution before each trial. The visual behavior experimental procedure and placement of objects is illustrated in Figure 2.

### Assessment of Retinal Morphology Using Haematoxylin and Eosin Staining

To evaluate the extent of retinal damage, the tissue morphology of retinal sections was examined by using H&E staining under a light microscope (×20 magnification), followed by quantitative morphometric analysis. All retinal sections were taken at 1 mm from the temporal edge of optic disc as previously described (Fazel et al., 2020). The morphometric parameters estimated in this study included the thickness of inner retina (IR) that comprised of inner plexiform layer and ganglion cell layer (GCL), the thickness of GCL, and the number of nuclei within IR as seen in the field of view. For morphometric analysis, the images were captured from three randomly chosen areas of each section and the measurements were performed assisted by an image analysis software, ImageJ (National Institutes of Health, Bethesda, MA, United States ). All measurements were performed by two independent observers and the average numerical values were taken as the final estimate. The morphometric measurements were used to calculate the fractional thickness of GCL within IR and the number of nuclei per 100 μm^2^ of IR (Takahata et al., 2003; Ohzeki et al., 2007; Razali et al., 2015; Razali et al., 2016; Lambuk et al., 2017; Mohd Lazaldin et al., 2018).

### Determination of Retinal 3-Nitrotyrosine Using ELISA

The measurement of 3-NT in the retinal samples allow indirect estimation of peroxynitrite indicating the level of nitrosative stress. Peroxynitrite is formed when nitric oxide reacts with superoxide radicals (Kang et al., 2020). The 3-NT ELISA assay (Elabscience) was performed in duplicates according to instructions provided by the manufacturer. The retinal tissues were homogenized in 100 µL of PBS on ice and centrifuged for 5 minutes at 5,000 x g to obtain the supernatant. All reagents and samples were brought to room temperature before use. Then 50 µL of standard working solution were added to the first two well columns in duplicate. Next, 50 µL of samples were then added to the other two well columns. Subsequently, 50 µL of Biotinylated Detection Ab working solution were added immediately to each well column. The plate was covered using the sealer provided with the ELISA kit and incubated for 45 min at 37°C. The solutions from each well column were aspirated, and 350 µL of wash buffer was added to each well, and left to soak for 1-2 min. When this was completed, the solutions from each well column were aspirated once again and patted dried against clean absorbent paper. These steps were repeated three times. 100 µL of HRP Conjugate working solution was then added to each well columns. The plate was covered using the provided sealer, then incubated once again for 30 min at 37°C. The solution from each well columns was aspirated and this washing process was repeated for five times. 90 µL of Substrate Reagent was added to each well column and the plate was covered using a new plate sealer. Following this step, the plate was incubated again for 15 min at 37°C. 50 µL of Stop Solution was then added to each well column and immediately, the optical density (OD value) of each well column was determined using a microplate reader (Victor X5, Perkin Elmer, United States) set to 450 nm.

### Statistical Analysis

Data were presented as mean ± standard error mean (SEM). Statistical comparison among groups was performed using one-way analysis of variance (ANOVA) with Turkey’s post-hoc test. *p* values of less than 0.05 was considered statistically significant.

## Results

### Effect of Philanthotoxin-343 on Exploratory Behaviour of N-methyl-D-aspartate-treated Rats in Open Field Test

**FIGURE 3:**
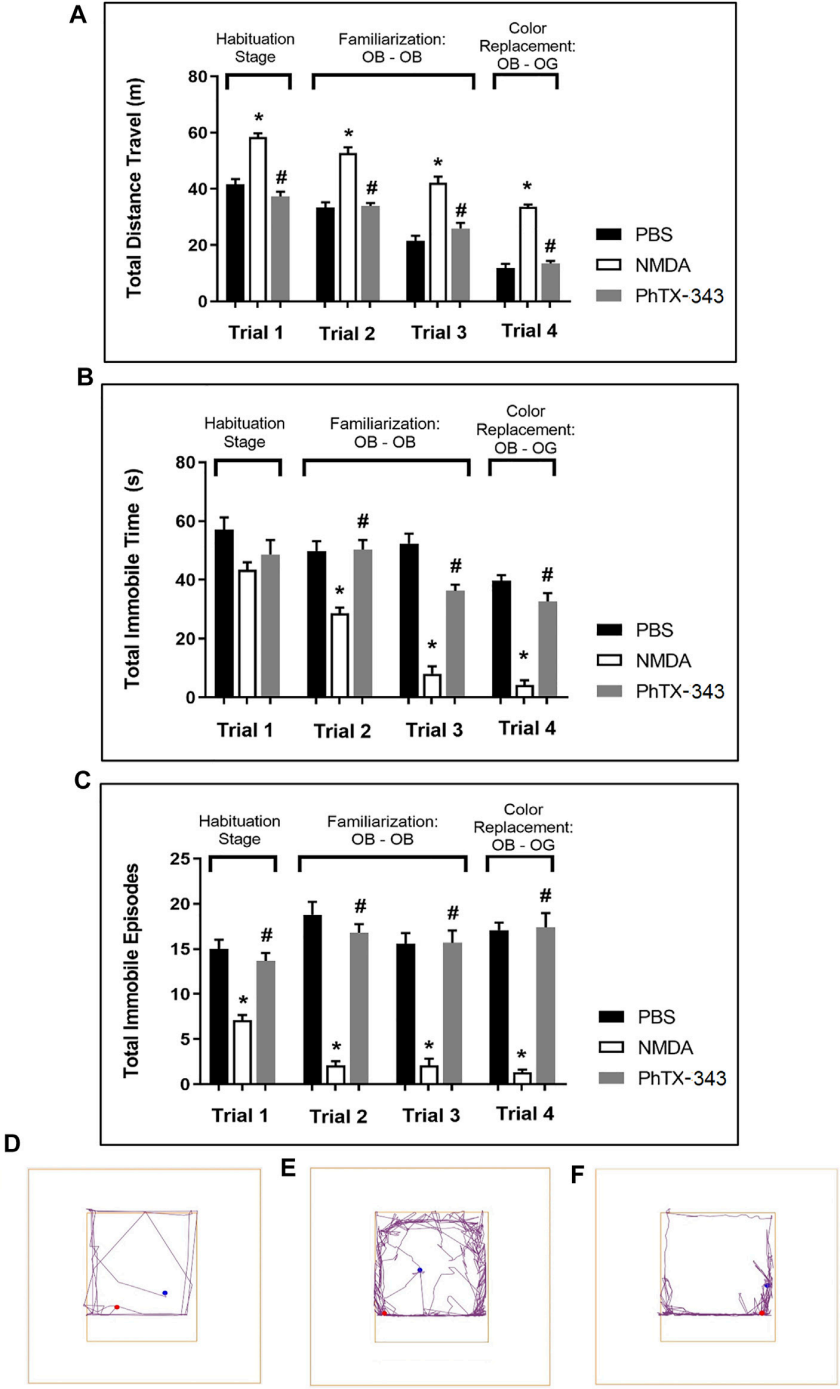
Effect of PhTX-343 on NMDA-induced changes towards exploratory and locomotor activities of rats in open field test. Bar graphs show **(A)** Total distance travelled, **(B)** Total immobile time, and **(C)** Total immobile episodes. Representative track plots for each group are shown in panel **(D)** PBS group, **(E)** NMDA group, and **(F)** PhTX-343 pre-treatment prior to NMDA exposure. *p < 0.05 vs PBS; #*p* < 0.05 vs NMDA. PBS, phosphate buffer saline; NMDA, N-methyl-D-aspartate; PhTX-343, philanthotoxin-343.

In the habituation stage of the open field test (trial 1), NMDA-treated rats travelled a significantly greater distance compared to PBS and PhTX-343 pre-treated rats (*p* < 0.05). The total immobile episodes in the NMDA group were significantly lower compared to PBS and PhTX-343 groups (*p* < 0.05). The total immobile time by NMDA-treated rats recorded higher mean values compared to both PBS and PhTX-343 pretreated rats, although the difference did not reach a significant level (*p* > 0.05). In trials 2 to 4, the total distance travelled was significantly higher for the NMDA group compared to both PBS and PhTX-343 groups (*p* < 0.05). Additionally, the NMDA group showed significantly lower total immobile time and total immobile episodes in trials 2 to 4 compared to PBS and PhTX-343 groups (*p* < 0.05). The outcome for PBS and PhTX-343 treated rats were comparable for all trials (*p* > 0.05). Figure 3 exhibits the exploratory behaviour of rats in open field test.

### Effects of Philanthotoxin-343 on Colour Object Recognition in N-methyl-D-aspartate-treated Rats

**FIGURE 4:**
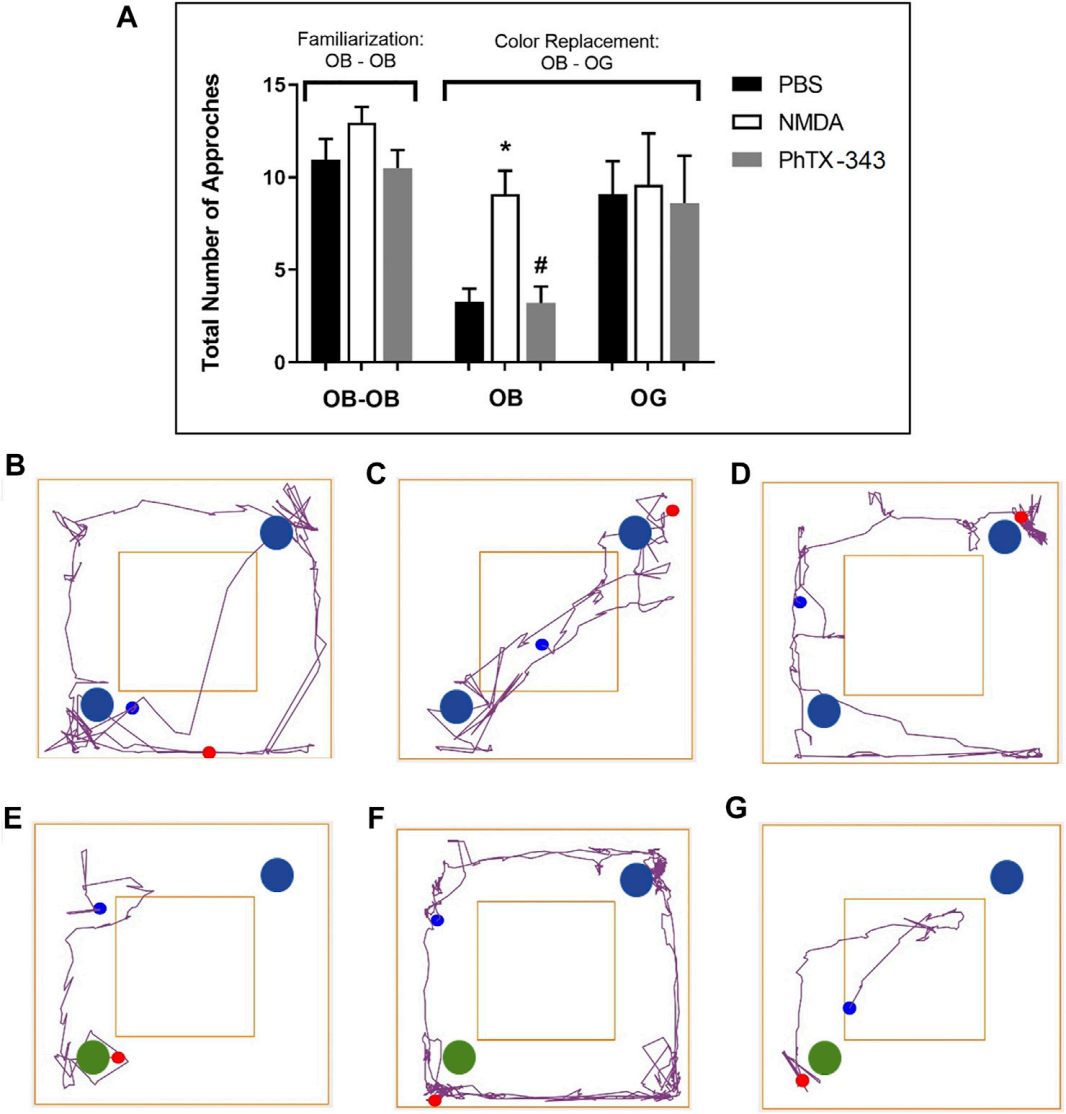
Effect of PhTX-343 on NMDA-induced changes in colour object recognition in rats **(A)** Bar graph showing the total number of approaches of rats at the colour objects; during familiarization stage. The open field arena contained two blue objects (OB1;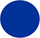 and OB2;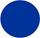), while during the colour replacement phase, one blue object (OB1;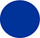) was replaced with a green object (OG;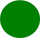). Representative track plots during familiarization stage for each group are shown in panel **(B)** PBS group, **(C)** NMDA group, and **(D)** PhTX-343 pre-treatment prior to NMDA exposure. Representative track plots during colour replacement phase for each group are shown in panel **(E)** PBS group, **(F)** NMDA group and **(G)** PhTX-343 pre-treatment group. *p < 0.05 vs PBS; #*p* < 0.05 vs NMDA. PBS, phosphate buffer saline; NMDA, N-methyl-D-aspartate; PhTX-343, philanthotoxin-343.

In the familiarization stage of the colour object recognition test as displayed in Figure 4, all rats showed almost similar pattern of activities in terms of the total number of approaches at the two blue colour objects (OB1;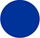 and OB2;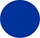). In the colour replacement test, when a novel green object (OG3;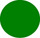) replaced one of the blue colour objects (OB1;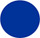), the remaining blue object was approached significantly much lesser by the PBS and PhTX-343 groups, 2.76- and 2.84-folds lower, respectively compared to the NMDA-treated rats (*p* < 0.05). Meanwhile the number of approaches at the novel green object (OG3;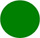) were similar among all groups (*p* > 0.05). Further analysis showed that while the NMDA group approached both objects similarly (*p* > 0.05) in spite of the colour object replacement, the PBS and PhTX-343 groups recorded significantly higher approaches at the novel green colour object (OG3;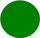) as opposed to the original blue colour object (OB2;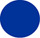) (*p* < 0.05).

### Effect of Philanthotoxin-343 on N-methyl-D-aspartate-Induced Changes in Retinal Morphology

**FIGURE 5:**
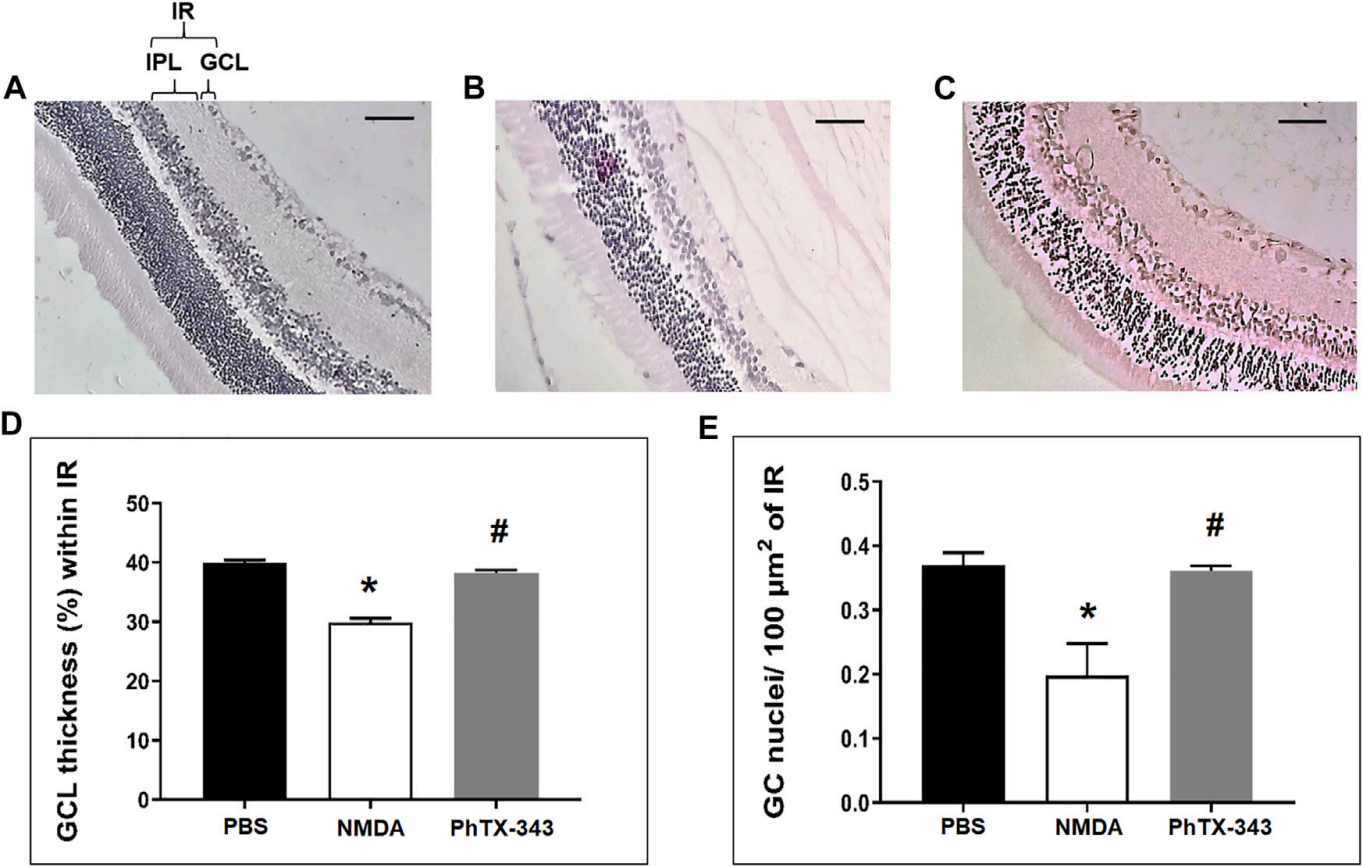
Effect of PhTX-343 on NMDA-induced changes in retinal morphology (×20 magnification) in rats. Photomicrographs (scale bar 100 µm) show H&E-stained retinal sections 7 days after intravitreal treatment for **(A)** PBS (control group), **(B)** NMDA-induced group and **(C)** Pre-treatment with PhTX-343, 24 h prior to NMDA administration. Morphometric quantification are presented as bar graphs **(D)** Fractional GCL thickness (%) within IR, and **(E)** Number of retinal cell nuclei per 100 µm^2^ of IR. The NMDA group demonstrated significant GCL thinning and lower retinal cell nuclei density compared to the PBS and PhTX-343 groups. Both analysis showed comparable results between the PhTX-343 and PBS groups. *p < 0.05 vs control; ^#^ *p* < 0.05 vs NMDA. PBS, phosphate buffer saline; NMDA, N-methyl-D-aspartate; PhTX-343, philanthotoxin-343, GCL, ganglion cell layer, IR, inner retina, IPL, inner plexiform layer.

As displayed in Figure 5, light microscopic examination of H&E-stained retinal sections in NMDA group showed relatively thinner GCL and IR compared to PBS-treated group. The density of nuclei within IR in NMDA-treated retinal tissues also appeared lesser compared to the PBS group. The rats that received PhTX-343 as pretreatment prior to NMDA exposure showed the thickness of GCL and IR, and retinal cell density that are comparable to that in the PBS group. Quantitative morphometric analysis showed that the fraction of GCL thickness within IR in NMDA-treated rats were lower than for PBS and PhTX-343 pre-treated groups by 1.34- and 1.28-folds, respectively (*p* < 0.05). NMDA-treated rats also showed significantly lower number of retinal cell nuclei/100 μm^2^ of IR compared to PBS and PhTX 343-treated rats, amounting to 1.87- and 1.82-fold differences, respectively (*p* < 0.05). Both parameters in PhTX-343-treated group were comparable to the PBS group (*p* > 0.05).

### Effects of Philanthotoxin-343 on N-methyl-D-aspartate-Induced Retinal 3-Nitrotyrosine Content

**FIGURE 6:**
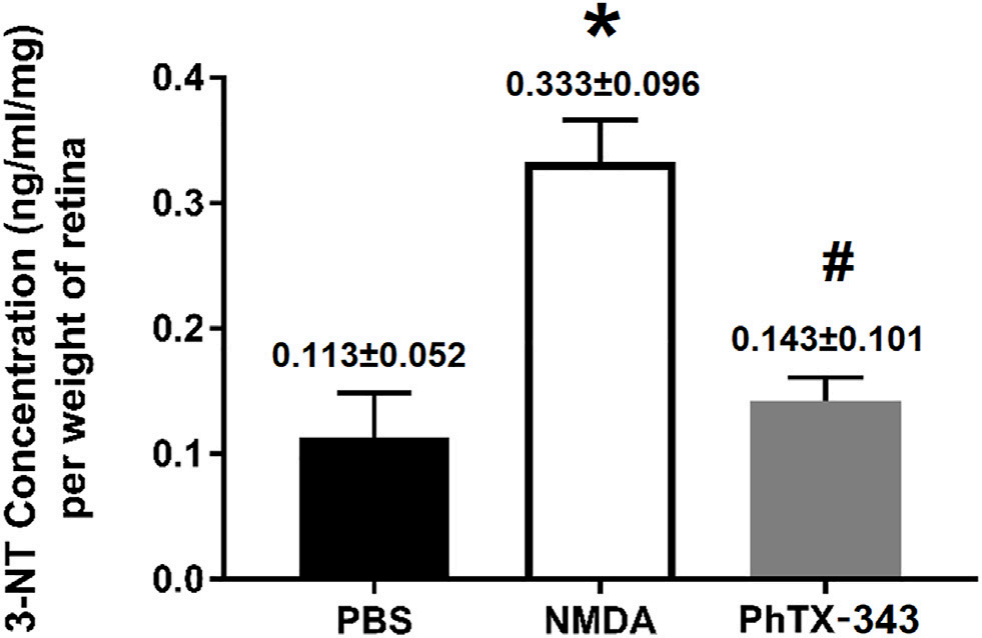
Effect of PhTX-343 7-days pretreatment on NMDA-induced retinal nitrosative stress in rats quantified by retinal 3-nitrotyrosine (3-NT) ELISA. The NMDA group had significantly higher 3-NT content compared to rats treated with PBS and pretreated with PhTX-343 prior to NMDA exposure. The 3-NT content for PBS and PhTX-343 groups were comparable. *p < 0.05 vs PBS; ^#^ *p* < 0.05 vs NMDA. PBS, phosphate buffer saline; NMDA, N-methyl-D-aspartate; PhTX-343, philanthotoxin-343.

As shown in Figure 6, the 3-NT content was significantly greater in NMDA-treated rat retinas compared to the PBS group amounting to a 1.76-fold difference (*p* < 0.05). The retinal 3-NT level in PhTX-343 pretreatment group was lower by 1.74-fold compared to the NMDA group (*p* < 0.05) and did not differ from the PBS group (*p* > 0.05).

## Discussion

This study provided evidence of the neuroprotective properties of PhTX-343 against NMDA-induced retinal cell loss in rats. We observed that pretreatment with PhTX-343 protects against NMDA-induced changes in the visual behaviour of rats, retinal morphology and 3-NT content. RGC death is the characteristic feature and pathological endpoint in glaucoma (Lambuk et al., 2017; Fazel et al., 2020; Mélik Parsadaniantz et al., 2020; Zollet et al., 2020). In glaucomatous eyes, overactivation of NMDAR by glutamate binding has long been proposed to result in excitotoxic retinal damage subsequent to excessive Ca^2^ influx into the ganglion cells (Chader, 2012; Huang et al., 2018). Accordingly, experimental studies in rodents have shown that NMDA exposure is associated with retinal injury (Bai et al., 2013; Niwa et al., 2016). Our findings demonstrate that the NMDA-treated rats show significant GCL thinning and reduced retinal cell density in IR, which is consistent with previous studies (Jafri et al., 2018; Lambuk et al., 2019b; Casado et al., 2019; Calvo et al., 2020; Fazel et al., 2020). Importantly, PhTX-343 pretreatment seems to abolish NMDA-induced changes in the retinal morphology as indicated by the significantly greater GCL thickness and retinal cell density in IR as compared to NMDA-treated rats. Since NMDA-induced retinal damage results from excessive Ca^2+^ influx and its consequences, it is logical to suggest that the protective effect of PhTX-343 is likely to be attributed to its ability to antagonize NMDAR. In fact, one of the *in vitro* studies using CA1 pyramidal neurons of rat hippocampus showed that PhTX-343 suppresses NMDA-gated currents, but did not affect other glutamate receptors (Fedorov et al., 1992). Other studies have also demonstrated that PhTX-343 is a non-competitive NMDAR antagonist (Green et al., 1996).

It is also noteworthy that NMDA-mediated excitotoxicity is associated with increased oxidative stress (Rokicki et al., 2015; Jafri et al., 2018). Nitrosative stress, which refers to the toxic condition caused by NO and reactive nitrogen species (RNS) overload, is a form of oxidative stress particularly implicated in neuronal excitotoxicity (Pacher et al., 2007; Pollard et al., 2016; Tatarkova et al., 2016; Papaefthymiou et al., 2020). NO excess and/or RNS formation (e.g. peroxynitrite (ONOO^−^)) affects mitochondrial function leading to the disruption of neuronal cell metabolism and survival. In combination with nuclear events, this condition promotes the pathogenesis of many neurodegenerative diseases including glaucoma (Ghasemi et al., 2018; Papaefthymiou et al., 2020). Estimation of 3-NT is an indirect measure of the peroxynitrite content (Luthra et al., 2005), and hence it was measured in the present study to assess the ability of PhTX-343 in suppressing nitrosative stress in NMDA-exposed retina.

We observed that NMDA exposure results in marked increase of 3-NT level in rat retinas compared to the healthy group. Pre-treatment with PhTX-343 appears to protect against NMDA-induced surge of retinal 3-NT level. Similar observations have been reported by earlier studies in response to NMDA exposure (Al-Gayyar et al., 2010; Ganapathy et al., 2011). Studies have shown that the elevation of NO level in rat retinal tissues after intravitreal injection of NO caused depletion of RGC, significant nuclear density reduction in GCL, and a half-life-dependent thinning of the inner plexiform layer (Jafri et al., 2018). Nitrosative stress can be further aggravated by oxidative stress resulting from excitotoxicity, whereby the excessive oxygen-free radicals (O^2−^) interact with NO yielding highly reactive peroxynitrite (Forder and Tymianski, 2009; Jafri et al., 2019). In the present study, pretreatment with PhTX-343 resulted in significant lowering of retinal 3-NT level indicating suppression of nitrosative stress. Since PhTX-343 is known to antagonize NMDA-mediated excitotoxicity (Fazel et al., 2020), it is likely that its effects on retinal morphology are a consequence of reduced production of NO in retinas, resulting in reduced nitrosative stress, and hence lesser retinal damage. Since preventing oxidative stress may add to the neuroprotective effects of PhTX-343, it remains to be determined whether it also suppresses the production of O^2-^ radicals or increases their removal (Jafri et al., 2019).

The corresponding changes in visual performance due to NMDA-induced retinal damage and the effects of PhTX-343 treatment were assessed by using visual behaviour tests in open field arena. The behavioural performance of rats in the presence of stress-inducing factors were assessed by observing exploratory and locomotor activities, which may be reflective of their visual abilities (Fazel et al., 2020; Jafri et al., 2020; Mohd Lazaldin et al., 2020; Lambuk et al., 2021). Our results clearly demonstrated that the NMDA group had trouble in navigating their way, which could be attributed to emotional and stress-associated responses, caused by visual impairments due to NMDA-induced excitotoxic damage (Fazel et al., 2020; Jafri et al., 2020; Mohd Lazaldin et al., 2020; Lambuk et al., 2021). In rats that received PhTX-343 pretreatment prior to NMDA exposure, the locomotor and exploratory behaviour of rats were comparable to that of the control group. Accordingly, both the PhTX-343 and control groups travelled significantly shorter total distances compared to the NMDA group, indicating relative comfort of animals in the surroundings. Similarly, total immobile time and episodes in both PhTX-343 and control groups were comparable. Significantly greater total distance travelled, and lesser immobile time and episodes by rats from the NMDA group indicate anxiety and stressful behaviour, which could be attributed to poor vision of rats, making them unable to see the surroundings, and hence raising the anxiety and stress levels. Similar effects of NMDA on rat behaviour after NMDA exposure have previously been reported (Jafri et al., 2020).

In the colour recognition test, the tasks involved rely on the innate preference of a rodent for novelty of different colour objects (Ennaceur and Delacour, 1988; Ennaceur, 2010; Warburton and Brown, 2015; Jafri et al., 2020). Failure of animals to recognize the replaced object with a different colour due to visual loss makes them recognize all objects as novel each time they are exposed to them (Ennaceur, 2010). It is important to note that rat vision is poor, where 97% of the retina are made up of rods (low-light vision), while the rest are made up of cones (daylight and bright-colour vision) (Ennaceur, 2010). Therefore, carefully selected shapes, colours and brightness of objects are paramount. In this study, blue (OB1;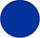 and OB2;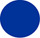) and green colour objects (OG3;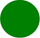) with a distinct shape were used as previously described (Jacobs et al., 2001; Ennaceur, 2010; Jafri et al., 2020). It was reported that rats can distinguish between ultraviolet and visible light, in the blue-green range.

In the colour object recognition test, the number approaches at each colour object during exploration in the arena were recorded. During the familiarization phase, the number of approaches at the two blue colour objects were similar for all groups. When one blue colour object was replaced by a green object, the rats in the control and PhTX-343 groups approached the green object significantly more in contrast to the original blue object, whereas rats in the NMDA group approached both objects equally. Additionally, the PhTX-343 and control groups were found to approach the original blue object significantly lesser compared to the NMDA group. This part of the study clearly showed that the control group and rats receiving PhTX-343 were able to “visualize” and “remember” the experiences from the familiarization stage and preferentially explore the novel introduced object relatively more than the original object it had replaced (Stefanko et al., 2009; Fazel et al., 2020; Jafri et al., 2020). The NMDA-treated rats, however, showed difficulties to recognize the colour objects and approached them equally, which is likely due to retinal cell injury resulting in loss of visual functions.

## Conclusion

In conclusion, our study demonstrated that pretreatment with PhTX-343 in rats suppressed NMDA-induced retinal nitrosative stress, and hence protected against retinal morphological alterations and preserved visual functions.

## Data Availability

The original contributions presented in the study are included in the article/Supplementary Material, further inquiries can be directed to the corresponding author.
